# On the Remote Cause of Epidemic Diseases

**Published:** 1842-01-01

**Authors:** 


### ON THE REMOTE CAUSE OF EPIDEMIC DISEASES. By *John Parkin*, Honorary and Corresponding Member of various learned Societies, &c. Octavo, pp. 198. Hatchard, 1841.

WITH laudable zeal, a philosophic mind, and unwearied industry, Mr. Parker prosecuted the investigation of the late epidemic cholera, both in this country and in Spain; and has now, after a considerable interval, given us the fruits of his meditations and reflections. We agree with the author that it is not in the moment of alarm, in the midst of an epidemic, that we can calmly trace its causes and ascertain its laws. But when is our profession free from an *emeute*, or some topic of contention that absorbs all its faculties for the day? We very much doubt whether one person in one hundred, of the medical practitioners of this country, could tell in what year the cholera invaded our shores! That terrible epidemic is as completely obliterated from their thoughts—we had almost said their *memory*—as though it had never existed! The reforms of colleges, the remuneration of workhouse doctors, the intrusion of chemists, and such-like topics, engross all attention, and we very much fear that Mr. Parkin will not be able to interest one medical man out of every thousand, so as to induce him to travel with him through the wilds of "remote causes," and epidemic influences. The profession is too much busied with the work of ascertainiug *effects*, to trouble themselves with the exploration of *causes*. Few of them have any relish for that kind of felicity which was promised by the Roman Bard to those who searched out and discovered the occult causes of things.

"Felix qui potuit RERUM cognosccre CAUSAS."

Now if Mr. Parkin has, or even thinks he has, made a discovery of hidden causes not hitherto unveiled, he already has his reward, according to the above precept of the poet, and may laugh at the neglect or scepticism of his contemporaries.

Our author believes, and we quite agree with him, that "the precise specific cause of the disease (cholera) remains entirely unknown, in spite of all the hypothetical opinions which have been put forth on the subject." It is however, something to know and confess our ignorance. He who has optical illusions is sure to take a false route, while the blind man uses every precaution, and proceeds in the most circumspect manner at every step.

"Entertaining a theory at variance with and different from those hitherto broached, and believing that it explains, not only all the facts bearing on this important subject, but, also, the various anomalies which belong to all other and acknowledged theories, I am induced to come forward on the present occasion, in order to make known my opinions, and to advance a few arguments in support of their truth." 8.

It is not very easy to get at the author's theory. One thing is certain, viz. that he does not ascribe the origin of cholera or any other epidemic to contagion.

"For the facts which have been presented to our notice, during the prevalence of the epidemic cholera, have set every conclusion drawn from the doctrine of contagion altogether at defiance; while they have, at the same time, shown that the premises upon which this doctrine is founded are false and untenable." 11.

It is quite unnecessary to go over the grounds by which the non-contagious character of cholera has been so often and so steadily maintained in this Journal. Several of the arguments and proofs, however, are cleverly brought forward in the present essay, and some of them set in a new light. One short extract from this long chain of argument, is all we can spare room for.

"It is no less a fact, that the epidemic commenced in the centre of France, and before any of the towns on the frontiers had been attacked: while it was impossible to refer the origin of the disease in Paris to the least communication with an infected town, or with infected individuals. Its simultaneous appearance, in fact, among numbers of individuals at the same moment, and in that class of persons who had the least intercourse with strangers, plainly showed that the doctrine of contagion could never account for the origin of the disease in that capital. 'We unhesitatingly avow our conviction,' remarks the editor of the *Lancet*—a work, be it observed, that had previously advocated the doctrine of contagion, 'that it would be worse than frivolous to discuss the proposition, that some other influence than contagion was concerned—and mainly concerned in the excitement of the disease in the French capital; and has since contributed powerfully and fatally to its propagation.'" 16.

If contagion then was not the cause of cholera, was it a poison, sui generis, rising from the earth, and inhaled into the lungs? Mr. Parkin observes, and justly, "that many of the phenomena presented during the march of epidemic diseases, are also common to the effects produced by the invisible but well-known agent, MALARIA." But if the two poisons be the same, the old doctrine, he remarks, of malaria or marsh poison being the product of decaying animal and vegetable matters, must be given up; since the cholera, for example, prevailed in the most barren and sandy tracts, as well as in the most luxurious and fertile localities.

The author's theory begins to dawn at page 34 of the Essay, in the following passage.

"If unable to account for the production of the poison above the surface, our only resource is, to glance into the interior of the globe, with the view of ascertaining whether there is any process going on there capable of giving origin to a poisonous matter. Now there is a process in constant operation in the bowels of the earth, and which gives rise, at particular periods, to certain effects cognizable to our senses: to this process the term *volcanic action* has been applied. But then it so happens, that this process is a silent and invisible one; for we are unable to penetrate into the interior of the globe, and view the operations of nature in this her hidden laboratory. It is impossible, therefore, to ascertain its existence, except by the occurrence on the surface of some of those phenomena, known to be produced by volcanic action. The principal and the most striking of the effects, directly produced by the agency of this cause, are, as is well and commonly known, the volcano and the earthquake." 35.

But, as volcanos and earthquakes do not always, or even generally accompany epidemics, we must extend the range of their cause (subterranean fires) far beyond the two phenomena when openly apparent to our senses. The writer of the article Geology, in the Encyclopædia Metropolitana, makes the following remark:—

"If we limit our view of volcanic action to the phenomena attendant on the eruption of a volcano, and the shock of the earthquake, we exclude from our definition a series of effects evidently allied to the former, and, perhaps, equally illustrative of its real nature. How different, for example, are the eruptions of Vesuvius and Ætna, or any other mountain which emits a stream of lava, or melted matter, from the emanations of gas and vapour, which arise in situations where no vent exists, or from the increased temperature of certain springs in the neighbourhood of active and extinct volcanos, or the evolution of carbonic acid, and other gases, from the water of these as well as all other thermal springs. Yet the connection of all these phenomena with the action, which gives rise to the eruption of the volcano and the discharge of melted matter from the crater, is as well established now, as is the relation of subterranean concussions or earthquakes with the volcanic process." 37.

Mr. Parkin, however, abandons this argument, as incapable of direct application to the cause of epidemics, whilst he tries to explain the connection of subterranean fire with this same cause by other ratiocination.

"Now, if we generalize the phenomena attendant on the march of epidemics, we shall find that they are so regular and uniform, as to deserve to be set down as laws of the disease. More than this, if we compare these laws with those attendant on volcanic action, we shall find that they are the same, or similar, as will be apparent by the recital of a few of the principal phenomena observed during the operation of this process on the crust of the globe.

THE FIRST AND MOST SINGULAR LAW WHICH MAY BE NOTICED, IS

THAT WHICH CAUSES THE EFFECTS OF VOLCANIC ACTION TO BE FELT OR WITNESSED ALONG PARTICULAR LINES OF THE EARTH'S SURFACE.

To be convinced of this, we have only to cast our eyes over any one of the principal volcanic regions, when we shall remark, that a series of vents extends along, at no great distance from each other, either in a straight or curvilinear direction; and this too over considerable portions of the earth's surface. As an example of the first, we may refer to the Andes, where, from Chili to the north of Mexico, there is a line of volcanos, *so uninterrupted*, that it is rare to find an intervening degree of latitude, in which there is not an active vent; and it seems probable, that they will hereafter be found to extend from Cape Horn to California, or even, perhaps, to New Madrid, in the United States—a *distance as great as the pole from the equator*.* Although extending to this distance in one continuous and uninterrupted line, the volcanic action, as well as the effects resulting from it, is confined to very narrow boundaries on either side. 'In regard to the eastern limits of this region,' observes the same writer, 'they lie deep beneath the waves of the Pacific, and must continue unknown to us.' On the west they do not appear to be prolonged to a great distance, for there seems to be no indications of volcanic disturbance in Guinea, Brazil, and Buenos Ayres.

'A remarkable example of the other variation or curvilinear direction, is to be found in the Pacific Ocean. From the Phillippine Islands, a range of volcanic vents proceeds to nearly 10° latitude, ranges westward along this parallel for about 25° of longitude, and then turns up north-west diagonally through about 125° of latitude. This line, which, when represented on maps, resembles an enormous fish-hook, passes from the Phillippines, by the north-east point of Celebes, Gelolo, the Volcanic Isles between New Guinea and Timor, Floris, Sumbawa, Java, and Sumatra to Barren Island.'

The paroxysmal convulsions, and other signs of internal action along these particular lines, and the fact, that two vents are seldom in a state of activity at the same time, while the discharge of matter from one outlet, invariably lessens, or arrests, that from another, sufficiently attest their continuity beneath the surface. Thus the volcanos in different parts of Iceland, as well as those in the Phlygrœan Fields, are observed, as Lyell states, to be in activity by turns,—one *vent often serving for a time as a safety valve to the rest.* Another proof, also, of the connection of certain volcanic vents, may be adduced from the fact that when several cones are thrown up in one eruption, which is sometimes the case, they invariably take a linear direction.

The principal volcanic region in the old world extends from east to west for the distance of about 1,000 miles from the Caspian Sea to the Azores. From south to north it reaches from about the 35th to the 45th degree of latitude. Its western limits, says Lyell, are the ocean, but it is impossible to ascertain how far it may be prolonged in that direction; neither can we assign with precision its extreme eastern boundaries, since the country beyond the Caspian, and sea of Arat, is scarcely known.

An attentive consideration of the phenomena which have been observed in this part of the world, from time to time, leads distinctly to the conclusion, that the volcanic action extends along the centre of this region in a line from east to west; for while the effects of earthquakes, which have occurred at a given point, have been felt hundreds of miles from the centre of concussion, in *a linear or western direction*, scarcely any effect has been observed in places situated but a comparatively short distance to the north or south of this particular line. This phenomenon was particularly noted in the earthquake, at Lisbon; for the concussion was severely felt by ships at sea, hundreds of miles to the westward of the spot where it first commenced; while places but slightly removed from this line to

* Lyell's Geology.

the north and south experienced no shock, but only slight agitation in the waters of the sea, rivers, ponds, etc. It would also be an easy task to show, if space were afforded for the purpose, that the concussions which have been felt in this region, and even at the farthest extremities of the volcanic boundaries have an intimate connection with the volcanos of Ætna and Vesuvius, inasmuch as for some time previous to an eruption of these mountains, earthquakes have generally been experienced along some portion of this particular line, and which have invariably ceased, as soon as the melted matter has found its way to the surface." 43.

Mr. Parkin remarks that when a shock has been experienced at a given spot, it is speedily propagated to another and distant point—" *always along some particular and well-defined line.*"

In the epidemic diseases under consideration a characteristic feature is progression along particular lines, whilst their effects extend but a short distance on the sides of these lines, like the volcanic shocks themselves. The line of cholera presented this peculiarity in a remarkable degree—attacking the inhabitants of one bank of a river, and sparing the other. It was the same with the " BLACK DEATH" of the 14th century. But we are unable to accompany our author through all the analogies between volcanic and epidemic phenomena. They evince great research after facts, and considerable ingenuity in harnessing these facts to the car of his theory. Thus, coincident with the " black death," a series of terrestrial commotions almost unexampled, occurred. An earthquake took place near Kingsai (where the disease first broke out) by which whole mountains were overturned, and a lake of more than a hundred leagues in circumference was formed. These concussions recurred for several years. The disease spread in a westernly direction across the continent of Asia to the shores of the Black Sea—to Constantinople—the cities of Europe—and ultimately to England—storms, floods, and earthquakes attending the route of this dreadful malady.

In respect to cholera, the Bengal Report states that, at first, the disease raged simultaneously in various and remote quarters:—

" But soon after reaching the junction of the Jumma and the Ganges, the epidemic began to show one of the most striking peculiarities, which characterized its march. It no longer pushed its influence, without distinction or apparent choice, in all directions and throughout every tract coming in its way; but began to affect *particular lines*, and to fix itself in particular divisions of the country, wholly restricting itself for the time to the course of those lines and divisions." 128.

Although it branched off occasionally to right and left, still the grand march was westerly till it reached England—crossed the Atlantic, and visited America!

Earthquakes are very rare in India; but about the time of the breaking out of the cholera there were several shocks felt in Bengal, and for some years afterwards. Thus at Bhooj, in 1819, there were 15,000 houses reduced to ashes in two minutes. But we can go no farther. Mr. Parkin thinks he has proved, or rendered it probable, that " various gases are not only generated in subterranean reservoirs, but are also extricated in considerable quantities into the surrounding atmosphere, and that to the direct action of some one or more of these products on the human frame we may possibly refer the production of epidemic diseases." Granting this—which however, we think is not very clearly proved—we are still in ignorance of the nature of this gas—or of the cause of its producing cholera at one time—fever at another—influenza at a third—and death, whether "black" or "blue," at all times. That the cause of cholera and other epidemic diseases is an emanation from the earth, and merely diffused in the air, we firmly believe, and have always maintained in this Journal. But that it is essentially connected with volcanic action, we are as yet far from being convinced, notwithstanding the ingenuity and industry brought to bear on the investigation by our author. A perusal of the work, however, will well repay the time expended, in consequence of the very curious information collected into a small volume from various and scattered sources.

